# Biosensors with Boronic Acid-Based Materials as the Recognition Elements and Signal Labels

**DOI:** 10.3390/bios13080785

**Published:** 2023-08-03

**Authors:** Lin Liu, Xiaohua Ma, Yong Chang, Hang Guo, Wenqing Wang

**Affiliations:** 1 College of Chemistry and Chemical Engineering, Anyang Normal University, Anyang 455000, China; 2 Henan Key Laboratory of Biomolecular Recognition and Sensing, Shangqiu Normal University, Shangqiu 476000, China

**Keywords:** boronic acid, molecular recognition, biosensors, ribonucleic acid, glycoprotein, cell imaging

## Abstract

It is of great importance to have sensitive and accurate detection of *cis*-diol-containing biologically related substances because of their important functions in the research fields of metabolomics, glycomics, and proteomics. Boronic acids can specifically and reversibly interact with 1,2- or 1,3-diols to form five or six cyclic esters. Based on this unique property, boronic acid-based materials have been used as synthetic receptors for the specific recognition and detection of *cis*-diol-containing species. This review critically summarizes the recent advances with boronic acid-based materials as recognition elements and signal labels for the detection of *cis*-diol-containing biological species, including ribonucleic acids, glycans, glycoproteins, bacteria, exosomes, and tumor cells. We also address the challenges and future perspectives for developing versatile boronic acid-based materials with various promising applications.

## 1. Introduction

In terms of sensitivity and selectivity, the recognition element is an important component of biosensors for the detection of biological-related substances, such as amino acids, nucleosides, glycans, proteins, antibodies, nucleic acids, exosomes, and cells [1,2]. The specific recognition of these species is classically based on antigen-antibody and aptamer-target interactions [3]. However, these bio-recognition elements are always expensive and require demanding storage and detection conditions. Therefore, scientists have devoted much effort to developing effective artificial/synthetic receptors in place of the biological recognition elements [4]. Among them, boronic acids have attracted considerable interest due to their specific sugar-responsive properties [5]. The principle of molecular interactions in boronate-affinity materials is the reversible covalent reaction between boronic acid ligands and *cis*-diol groups to form five- or six-membered cyclic esters in an alkaline aqueous solution [6]. The change of the surrounding pH into acid and the presence of other *cis*-diol-containing compounds can dissociate the boronate esters, leading to the release of the captured targets.

Boronate-affinity materials were primarily employed to immobilize and isolate *cis*-diol-containing compounds ten years ago in combination with traditional detection techniques [7,8,9,10]. For instance, boronic acid-modified columns and beads have been used to enrich glycoproteins for mass spectroscopy-based glycoproteome analysis [11,12,13,14]. Meanwhile, boronic acid derivatives with fluorescent or electrochemical properties are used to detect small *cis*-diol-containing species through the boronate-affinity-induced signal change [15]. Additionally, boron compounds with Lewis acid features can react with nucleophiles such as fluoride ions (F^−^) and cyanide ions (CN^−^) [16,17,18,19]. Phenylboronate compounds can be oxidized into phenol by hydrogen peroxide (H_2_O_2_). Based on this phenomenon, boronic acid derivatives have been utilized to determine F^−^, CN^−^, H_2_O_2_, and carbohydrates (e.g., glucose, fructose, and galactose) [20,21,22,23,24]. The analytical properties of boronate-affinity materials (e.g., pH stability, binding affinity, and sensing selectivity) are dependent upon the structure of boronic acid ligands. When the surrounding environmental pH is greater than the pKa, boronic acid in the form of a tetragonal boranate anion (sp^3^) can combine with *cis*-diol to produce five or six-membered cyclic eater bond. When the pH is acidic, the complex is decomposed because of the limited binding between *cis*-diol and boronic acid in a trigonal configuration (sp^2^) [25,26]. Nevertheless, the high binding pH is unfavorable for the actual physiological detection application. To improve the sensing performances, the fundamental issues have been widely investigated and addressed in the past decade [27]. For example, three types of boronic acidderivatives have been reported to exhibit low pH-binding ability and successfully used to prepare boronate-affinity materials, including electron-withdrawing groups-derived, Wulff-type and heterocyclic boric compounds [28,29,30,31]. The interactions between different boronic acidderivatives and *cis*-diol-containing molecules have been investigated by various analytical methods, such as affinity capillary electrophoresis and surface plasmon resonance (SPR) [32,33,34]. Currently, a broad variety of boronate-affinity materials (e.g., macroporous monoliths, self-assembled materials, mesoporous materials, nanoparticles, molecularly imprinted polymers (MIPs), and polymer brushes) have been developed for the immobilization and separation of various *cis*-diol-containing compounds [35,36,37,38,39,40,41,42,43,44,45]. Especially boronate-affinity-oriented surface imprinting methods have been well used in the isolation and immobilization of glycopeptides and glycoproteins because of their attractive features, such as excellent specificity, enhanced binding strength, widely applicable binding pH, and good tolerance for non-target *cis*-diols [46,47,48,49,50,51].

Nowadays, boronic acid derivatives and boronate-affinity materials have been widely used in various applications, such as targeted drug delivery, drug discovery, catalysis, and biosensing [52,53,54,55,56,57]. With the advancement of nanotechnology and bioanalytical techniques, nanomaterials functionalized with boronic acid groups have been widely utilized to construct different methods for the detection of *cis*-diol-containing biological-related substances, including ribonucleic acids, glycans, glycoproteins, bacteria, exosomes, and tumor cells [58]. For example, Gao et al. reported an integrated surface-enhanced Raman scattering (SERS) platform for the detection of bacteria using boronic acid-modified plasmonic gold film to capture bacteria [59]. As one type of glycoprotein, antibodies with carbohydrate moieties in the constant domain Fc can be immobilized on the surface of boronic acid-modified substrates or nanomaterials in a site-specific and self-oriented manner [60,61,62]. Moreover, versatile techniques combined with nanomaterials have been applied to the dynamic and real-time imaging of glycosylation and glycoproteins on living cells, such as fluorescence microscopy, atomic force microscopy, and dark-field optical microscopy [63,64]. Boronic acid-based polymeric hydrogels have been synthesized to fabricate glucose-responsive magnetic acoustic resonance sensors or soft sensors for glucose-stimulated insulin release [65,66]. Moreover, with the fast growth of nanoengineering and biomimetic manufacturing, boronic acid-based materials and sensing devices have been constructed for reliable, user-friendly, non-invasive, and cheap healthy diagnostics and biomedical applications [67,68]. Several professional reviews in this area have been reported [9,69,70,71,72,73,74]. For example, the advances in boronic acid-based optical chemosensors were summarized [75,76,77]. Sun et al. reviewed the development of boronic acid derivatives for fluorescence imaging of carbohydrates [78]. Anzai et al. reported the progress in electrochemical biosensors based on phenylboronic acid derivatives [79]. Li et al. addressed the recent progress of boronic acid-based electrochemical sensors for the detection of biological analytes [80]. Liu’s group published several review papers about the applications of boronate-affinity materials for the separation and detection of *cis*-diol-containing compounds [81,82,83,84]. Recently, plenty of novel boronic acid-based molecules and nanomaterials have been exploited to develop different sensing platforms. In this work, we aim to give a comprehensive summary of the development of biosensors with boronic acid-based materials as recognition elements and signal labels. We focus on the roles of boronic acid-based materials in biosensor construction and classified this review into five parts according to the types of detection techniques.

## 2. Boronate-Affinity-Based Electrochemical Biosensors

Electrochemical biosensors have been widely used in environmental monitoring, food safety, and disease diagnosis because of their excellent properties, such as fast response, high sensitivity, and low cost. At first, different boronic acid derivatives with an electroactive moiety (e.g., ferrocene, viologen, and triphenylmethane) were elaborately synthesized for the development of sensors for saccharide detection [85,86,87]. The binding of them with saccharides led to a shift in oxidation potential [88]. Afterwards, boronic acid derivatives were utilized as synthetic receptors to modify the sensor electrodes for the capture of *cis*-diol target substances [89,90,91,92,93]. For example, Tang developed a nanopore-based single-entity electrochemical technique for label-free detection of single-molecule glycoprotein-boronate affinity [94]. Different nanomaterials have been used to modify the electrodes in combination with boronate-affinity interactions, enhancing the sensitivity of label-free electrochemical biosensors, such as gold nanoparticles (AuNPs) and graphene oxide (GO) [95,96,97]. Typically, Thiruppathi et al. developed a disposable electrochemical biosensor for the detection of glycated hemoglobin based on boronic acid derivative and multiwalled carbon nanotube-modified screen printed carbon electrode (SPCE) [98]. Hu et al. fabricated a biomimetic boronic acid-modified graphene-based 3D scaffold for cell culture and electrochemical monitoring [99]. The capture of the target *cis*-diol substance can inhibit electron transfer at the electrode/solution interface, resulting in a change in the electrochemical signal. However, the low discrimination ability of boronic acid toward 1,2- and 1,3-diol dramatically limits the selectivity of such methods against a range of saccharides and glycoproteins [100].

To avoid nonspecific binding and improve selectivity, many efforts have been devoted to the fabrication of sandwich-type biosensors, in which *cis*-diol-containing targets were captured by a recognition element-modified electrode and then recognized with boronic acid-modified signal labels, providing a detectable electrochemical signal. For instance, boronate-affinity controllable-oriented imprinting can be utilized for surface printing and molecular immobilization on the boronic acid-modified electrode [101]. In this part, we mainly summarized the progress of electrochemical biosensors with boronic acid-based electroactive molecules and nanomaterials as recognition elements and signal labels to produce electrochemical responses (Table 1).

### 2.1. Boronic Acid-Based Electroactive Molecules for Electrochemical Biosensors

**Figure 1:**
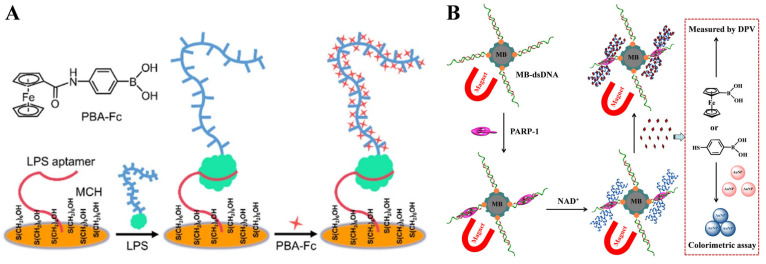
(**A**) Schematic illustration of the boronate-affinity-based amplified electrochemical LPS aptasensor [107]. Copyright 2022, American Chemical Society. (**B**) Schematic illustration of magnetic-based electrochemical and colorimetric strategies for PARP-1 detection by sequestrating FcBA or MPBA [108]. Copyright 2021, Elsevier.

Boronic acid modified with a redox-active moiety (e.g., ferrocene, viologen, and triphenylmethane) can be directly used as an electroactive label to recognize the *cis*-diol unit of the target that was captured by the recognition element-modified electrode [102,103]. For example, Hu and co-workers have reported several electrochemical biosensors for the detection of biomolecules using 4-(ferrocenylacetamido)phenyl)boronic acid (FcPBA) as the electroactive label, including glycoproteins, DNA, and lipopolysaccharides (LPSs) [104,105,106,107]. One of their works shown in Figure 1A is the electrochemical aptasensing of LPS through the boronate-affinity interaction [107]. LPS was captured by the aptamer immobilized on the electrode. The polysaccharide chain of LPS consisted of hundreds of *cis*-diol moieties, which allowed for the capture of electroactive FcPBA molecules via the formation of boronate ester bonds. The site-specific decoration of target LPS with hundreds of signal tags resulted in the generation of an amplified electrochemical signal. Compared with conventional enzyme-based methods, this method showed advantages of unrivaled simplicity, rapidity, and cost-effectiveness. In addition, our group developed magnetic bead-based electrochemical and colorimetric methods for the determination of poly(ADP-ribose) polymerase-1 (PARP-1) using ferroceneboronic acid (FcBA) and 4-mercaptophenylboronic acid (MPBA) as signal probes [108]. As illustrated in Figure 1B, after the capture of PARP-1 by the MB-dsDNA, auto-PARylation was triggered in the presence of *β*-nicotinamide adenine dinucleotide (NAD^+^). The formed poly(ADP-ribose) (PAR) polymer consisted of abundant ribose units and covalently adsorbed FcBA or MPBA through the formation of boronate ester bonds. The change in the level of FcBA or MPBA in solution could be readily determined by measuring the electrochemical signal of FcBA or by monitoring the MPBA-induced aggregation and color change of AuNPs. Similarly, circulating tumor cells and *Escherichia coli* with plenty of sugar units on their surfaces have been determined by electroactive boronic acid derivatives with the aid of magnetic nanomaterials [109,110].

**Figure 2:**
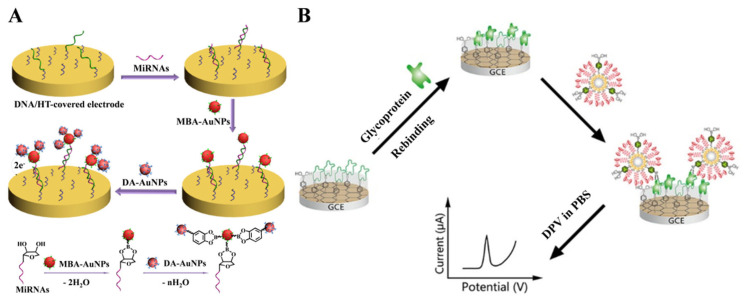
(**A**) Schematic illustration of the capture of miRNAs by the immobilized DNA probes and the detection of miRNAs by the attachment of MPBA-AuNPs and DA-AuNPs and the formation of boronate ester bonds between MPBA-AuNPs and miRNAs as well as DA-AuNPs [115]. Copyright 2013, Elsevier. (**B**) Schematic illustration of the fabrication of the boronate-affinity sandwich assay and the electrochemical detection of glycoproteins procedure [117]. Copyright 2017, American Chemical Society.

The limited number of sugar units on the targets will result in the capture of a small number of electroactive molecules, thus limiting the detection sensitivity. Nanomaterials with a high surface area can act as nanocarriers to load boronic acid derivatives and electroactive molecules, amplifying the electrochemical signals [111,112,113,114]. For this consideration, our group developed an electrochemical biosensor for miRNA detection based on the formation of boronate ester bonds and the dual-amplification of AuNPs [115]. As shown in Figure 2A, miRNA containing a *cis*-diol group at the end of the chain was labeled with MPBA-AuNP through the covalent interaction between boronic acid group in MPBA-AuNP and *cis*-diol in miRNA. Furthermore, dopamine (DA)-modified AuNPs (DA-AuNPs) were tethered to the surface of MPBA-AuNPs via the interactions between boronic acids and diphenols. A large number of electroactive DA molecules could produce an amplified voltammetric signal. The boronate-affinity-based dual-amplification strategy has also been applied to sandwich-like electrochemical detection of glycoproteins [116]. In addition, You et al. developed an electrochemical platform for horseradish peroxidase (HRP) detection based on boronate-affinity-based MIP, 6-ferrocenylhexanethiol (FcHT), and MPBA-modified SiO_2_@Au nanocomposites (SiO_2_@Au/FcHT/MPBA) [117]. As displayed in Figure 2B, after the capture of glycoprotein (HRP) onto the MIP film-modified electrode, SiO_2_@Au/FcHT/MPBA were added to react with the *cis*-diols in HRP, generating a strong electrochemical signal.

**Figure 3:**
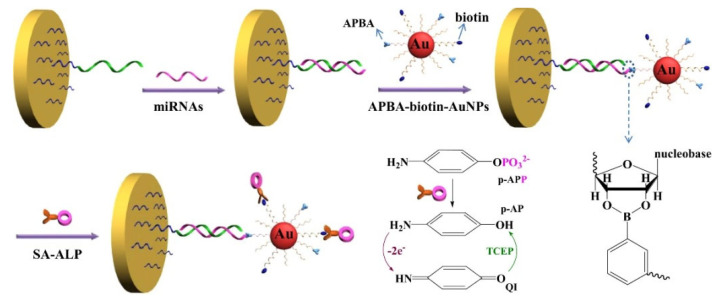
Schematic illustration of the label-free detection of miRNAs based on the triple signal amplification of APBA-biotin-AuNPs, SA-ALP and the *p*-AP redox-cycling reaction [119]. Copyright 2014, Elsevier.

Enzymatic reactions have been well combined with redox cycling for signal amplification [118]. Boronic acid derivatives and enzymes can be simultaneously loaded on the surface of nanomaterials. Our group developed an electrochemical biosensor for the detection of miRNAs based on the triple signal amplification of AuNPs, alkaline phosphatase (ALP), and the redox cycling reaction [119]. As shown in Figure 3, MPBA-biotin-AuNPs were used to label miRNAs through the formation of boronate ester bands. Furthermore, the streptavidin-conjugated ALP (SA-ALP) conjugates were attached to the biotin-peptide-modified AuNPs via the SA-biotin interactions. ALP could catalyze the hydrolysis of the electrochemically inactive substrate *p*-aminophenyl phosphate (*p*-APP) into the electroactive product *p*-aminophenol (*p*-AP). The oxidized *p*-AP could be cycled by the reducing reagent tris(2-carboxyethyl)phosphine (TCEP), thus enabling an increase in the anodic current.

### 2.2. Boronic Acid-Based Nanomaterials for Electrochemical Biosensors

**Figure 4:**
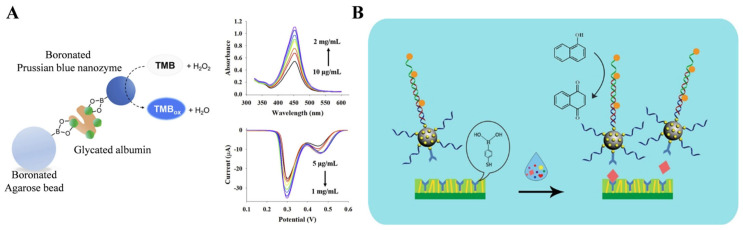
(**A**) Schematic illustration of the boronate-affinity sandwich assay for determination of glycated albumin based on APBA-modified Prussian blue nanazyme [124]. Copyright 2020, Elesiver. (**B**) Schematic illustration of the proposed sensing platform for electrochemical detection of ovalbumin by using SiO_2_@Au/dsDNA/CeO_2_ as the signal tag [125]. Copyright 2019, American Chemical Society.

Nanomaterials with enzyme-like characteristics (nanozymes) can be used as substitutes for natural enzymes to promote the sensing performance of electrochemical biosensors [120,121,122,123]. For this view, Son et al. developed a boronate-affinity sandwich bioassay for glycated albumin using 3-aminophenylboronic acid (APBA)-modified Prussian blue nanozymes (Figure 4A) [124]. In this study, glycated albumin was captured by boronated agarose beads and then labeled with APBA-modified nanozymes. After the formation of sandwich complexes, nanozymes catalyzed the oxidation of TMB by H_2_O_2_, and the oxidized TMB products were determined by differential pulse voltammetry (DPV). In addition, DNA techniques such as hybridization chain reaction (HCR), rolling amplification reaction, and strand displacement reaction can be integrated with electrochemical biosensors for signal amplification. Sun et al. developed an ultrasensitive microfluidic paper-based analytical device (μPAD) for the electrochemical detection of glycoprotein ovalbumin based on MIP film and boronate-affinity interaction [125]. As displayed in Figure 4B, SiO_2_ nanoparticles were sequentially modified with AuNPs, MPBA, and capture DNA (SiO_2_@Au/dsDNA). After the HCR reaction in the presence of two hairpin DNA strands, a large number of CeO_2_ NPs were linked to the dsDNA polymers through the amidation reactions (SiO_2_@Au/dsDNA/CeO_2_). The μPAD surface was decorated with Au nanorods and MPBA to form boronate-affinity-based MIPs with double recognition capacity. Ovalbumin was captured by MIPs and then labeled with SiO_2_@Au/dsDNA/CeO_2_ through boronate-affinity interactions. The redox-active CeO_2_ nanoparticles catalyzed the conversion of 1-naphthol to naphthoquinone, producing an amplified electrochemical signal.

**Figure 5:**
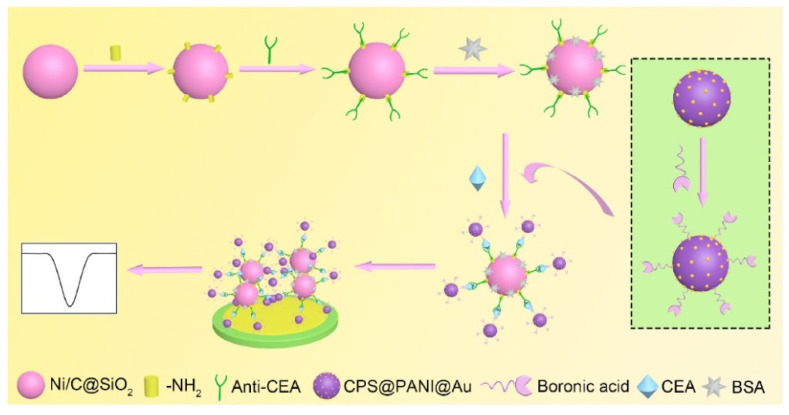
Schematic illustration of the procedures for the construction of sandwich-type electrochemical biosensors based on boronic acid-functionalized CPS@PANI@Au [127]. Copyright 2021, Elesiver.

Due to the high abundance of metal ions, nanomaterials can be electrochemically decomposed into metal ions in acid solution and then electrochemically reduced, producing a strong redox peak for signal amplification. In this aspect, Amor-Gutiérrez et al. used Ag_2_S QDs as electrochemical labels to determine *Escherichia coli* [126]. Song et al. reported a sandwich-type electrochemical immunosensor for carcinoembryonic antigen (CEA) detection using boronic acid-functionalized AuNPs-loaded nanocomposites as sacrificial labels [127]. As illustrated in Figure 5, magnetic hollow magneticsilica coated with nickel/carbon (Ni/C@SiO_2_) nanocomposites were modified with anti-CEA as the immune sensing platforms. CEA was captured and then recognized by boronic acid-functionalized CPS@PANI@Au. After the magnetic separation, the sandwich-type complexes were transferred onto the electrode surface. After 120 s of pre-oxidation in 0.1 M HCl solution at 1.25 V, AuNPs were oxidized to Au^3+^ ions and the reduction signal was measured by DPV.

**Figure 6:**
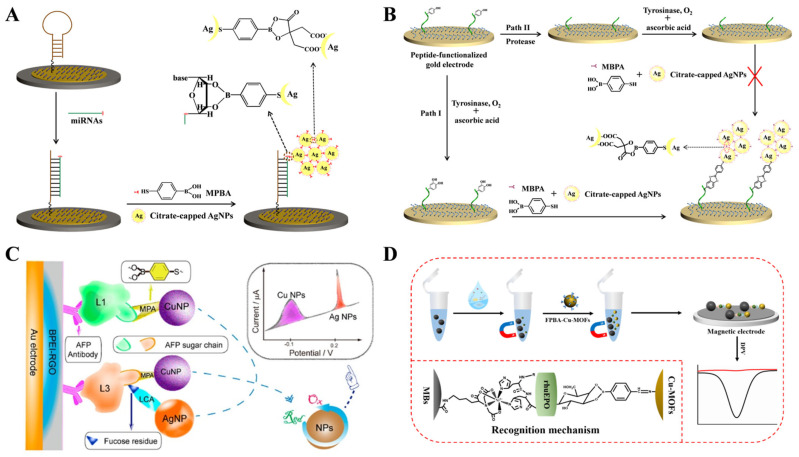
(**A**) Schematic illustration of the proposed electrochemical strategy for miRNA detection based on MPBA-induced in situ formation of AgNP aggregates as labels [130]. Copyright 2017, Elsevier. (**B**) Schematic illustration of the proposed electrochemical strategy for tyrosinase and protease detection based on MPBA-induced in situ formation of AgNPs aggregates as labels [133]. Copyright 2017, Elsevier. (**C**) Schematic illustration of the electrochemical bioassay for the determination of AFP-L3 in total AFP [134]. Copyright 2018, American Chemical Society. (**D**) Schematic illustration of antibody-free and enzyme-free electrochemical detection of recombinant glycoproteins using FPBA-modified Cu-MOFs as signal tags [138]. Copyright 2023, Elsevier.

Silver and copper nanoparticles (AgNPs and CuNPs) can be directly electrochemically oxidized to generate a well-definedredox peak [128,129]. Our group have developed several electrochemical biosensors for the determination of miRNAs and glycoproteins based on MPBA-induced in-situ formation of AgNPs aggregates for signal amplification [130]. As shown in Figure 6A, miRNAs captured by the DNA-modified electrode could be labeled with MPBA via the formation of boroante esters, and then the thiol group in MPBA could capture citrate-capped AgNPs through Au-S interaction. Meanwhile, free MPBA molecules in solution induced the in-situ assembly of AgNPs on the electrode surface via the covalent interactions between the α-hydroxycarboxylate of citrate and the boronic acid group of MPBA and the formation of Ag-S bonds. The oxidation of AgNP networks produced a significantly amplified electrochemical signal. The strategy based on MPBA-induced in-situ formation of AgNP aggregates on the electrode surface was further used to detect glycoproteins and enzymes [131,132]. For instance, Xia et al. reported electrochemical bioassays of tyrosinase and protease (thrombin) based on the MPBA-induced in-situ formation of AgNPs aggregates [133]. As displayed in Figure 6B, tyrosinase could catalyze the hydroxylation of tyrosine residue in the peptide modified on the electrode surface. Furthermore, the generated *o*-diphenolmoieties were labeled by MPBA/AgNPs, producing a strong electrochemical signal. However, thrombin could catalyze the cleavage of peptides, thus inhibiting the formation of MPBA/AgNPs networks on the electrode surface.

In contrast to noble metal nanoparticles, CuNPs are much cheaper, and their oxidation peak potential is well separated from that of AgNPs. Thus, CuNPs have been used as electrochemical tracers for the detection of targets. Wei et al. developed an electrochemical immunosensor for the determination of the alpha fetoprotein-L3 (AFP-L3) isoform ratio (AFP-L3%) using MPBA-Cu NPs and Lens culinaris agglutinin (LCA)-AgNPs as electroactive tags [134]. As illustrated in Figure 6C, after the capture of AFP, MPBA-Cu NPs were used to label the total AFP through the interaction between the boronic acid group and carbohydrate. LCA-AgNPs were used to specifically bind with the fucose of AFP-L3. Based on the independent oxidation signals of CuNPs and AgNPs, the concentrations of AFP and AFP-L3 have been simultaneously quantified. To improve the sensitivity and accuracy, An et al. developed a ratiometric electrochemical biosensor for the detection of exosomal glycoproteins using MPBA-modified core-shell nanoparticles of silica-silver (MPBA-SiO_2_@AgNPs) as signal labels [135]. SiO_2_ was used to carry the CD63 aptamer and N-(2-((2-aminoethyl)disulfanyl)ethyl) ferrocene carboxamide (FcNHSSNH_2_). The nanocomposites were immobilized on the surface of GO-cucurbit[7] (GO-CB[7])-modified electrode through the host-guest interaction between Fc and CB[7]. After the capture of exosomes by CD63 aptamers, MPBA-SiO_2_@AgNPs were introduced to identify glycoproteins on the exosome surface. A large amount of AgNPs produced an amplified electrochemical signal with FcNHSSNH_2_ as the internal reference molecule.

As a typical class of crystalline porous materials, metal-organic frameworks (MOFs) are self-assembled from metal ions/clusters and organic ligands. Through the elegant design of electroactive building blocks, MOFs can be directly used as signal probes in electrochemical bioassays for signal amplification [136,137]. Very recently, we reported an antibody and enzyme-free electrochemical biosensor for the determination of recombinant glycoproteins based on nitrilotriacetic acid-nickel ion (NTA-Ni^2+^)-modified magnetic beads (MBs-NTA-Ni^2+^) and 4-formylphenylboric acid (FPBA)-functionalized Cu-MOFs (FPBA-Cu-MOFs). As shown in Figure 6D, recombinant human erythropoietin (rhuEPO) was captured by MBs-NTA-Ni^2+^ through the sequential chemical recognition of NTA-Ni^2+^ and the hexahistidine (His_6_) tag on the target protein. After magnetic separation, FPBA-Cu-MOFs were attached to the target through the interaction between the boric acid group of PFBA and the glycan residue on rhuEPO. The magnetic sandwich conjugates were transferred onto the electrode with the aid of a magnet. A strong DPV signal was obtained from the electrochemical reduction of abundant Cu^2+^ ions in the MOFs.

**Table 1:** Analytical performances of various electrochemical biosensors with boronic acid-based materials as recognition elements and signal labels for the detection of ribonucleic acids, glycoproteins, bacteria, exosomes, and tumor cells.

Receptors	Target	Signal Label	Linear Range	LOD	Ref.
PNA	DNA	FcPBA	1 × 10^−5^–10 nM	2.9 fM	[104]
Aptamer	Mucin 1	FcPBA	5 × 10^−2^–50 U/mL	0.021 U/mL	[105]
Aptamer	LPS	FcPBA	1 × 10^−3^–1 ng/mL	0.34 pg/mL	[107]
dsDNA	PARP-1	FcBA	0.1–50 U	0.1 U	[108]
Aptamer	CTCs	FcBA	50–2 × 10^4^ cells	50 cells	[109]
IgY	*E. coli*	FcBA	10–10^8^ CFU/mL	3 CFU/mL	[110]
Aptamer	Thrombin	Fc-MMA-based polymer	5 × 10^−2^–100 pM	35.3 fM	[106]
Aptamer	CEA	MPBA/Thi/SiO_2_	1 × 10^−3^–10 ng/mL	0.49 pg/mL	[111]
Biotin	Avidin	Fc/MPBA-AuNPs	0.75–19.6 pM	0.2 pM	[112]
Aptamer	V.P	AuNPs/FcHT/MPBA	10–10^9^ CFU/mL	3 CFU/mL	[114]
DNA	miRNA-21	MPBA-AuNPs and DA-AuNPs	0.1–10 pM	45 fM	[115]
Aptamer	PSA	MPBA-AuNPs and DA-AuNPs	0.125–3.65 pM	50 fM	[116]
MIP	HRP	SiO_2_@Au/FcHT/MPBA	1 × 10^−3^–100 ng/mL	0.57 pg/mL	[117]
Aptamer	rHuEPO	MPBA-biotin-AuNPs and ALP	0.02–2 pM	8 fM	[118]
DNA	miRNA-21	APBA-biotin-AuNPs and ALP	0.01–5 pM	3 fM	[119]
APBA	glycated albumin	APBA-PBNPs	5 × 10^−3^–1 mg/mL	3.47μg/mL	[124]
MIP	OVA	MPBA-SiO_2_@Au/dsDNA/CeO_2_	1–1 × 10^6^ pg/mL	0.87 pg/mL	[125]
Antibody	CEA	BA-CPS@PANI@Au	6 × 10^−3^–12 ng/mL	1.56 pg/mL	[127]
Annexin-V	apoptotic Jurkat cells	ABA-AuNPs and silver	1 × 10^2^–3.5 × 10^3^ cells	38 cells	[129]
DNA	miRNA-21	MPBA and citrate-AgNPs	0.1–50 fM	20 aM	[130]
Aptamer	PSA	MPBA and citrate-AgNPs	0.5–200 pg/mL	0.2 pg/mL	[131]
Peptide	Tyrosinase and thrombin	MPBA and citrate-AgNPs	1 × 10^−3^–0.5 mU/mL and 0.025–5 ng/mL	0.1 nU/mL and 0.02 ng/mL	[133]
Antibody	AFP-L3 and total AFP	MPBA-CuNPs and LCA-AgNPs	50–1 × 10^5^ pg/mL and 0.4–1 × 10^3^ng/mL	40 and 10 pg/mL	[134]
Aptamer	Exosomal glycoprotein	MPBA-SiO_2_@Ag	4.2 × 10^2^–4.2 × 10^9^ particles/μL	368 particles/μL	[135]
NTA-Ni^2+^	rhuEPO	FPBA-Cu-MOFs	0.01–50 ng/mL	5.3 pg/mL	[138]

Abbreviation: PNA, peptidenucleic acid; FcPBA, (4-(ferrocenylacetamido)phenyl)boronic acid; Fc-MMA, ferrocenylmethyl methacrylate; LPS, lipopolysaccharide; dsDNA, double-stranded DNA; PARP-1, poly(ADP-ribose) polymerase-1; FcBA, (dihydroxyboryl)ferrocene; CTCs, circulating tumor cells; IgY, egg yolk antibody; MPBA, 4-mercaptophenylboronic acid; Thi, thionine; CEA, carcinoembryonic antigen; Fc, ferrecene; FcHT, 6-ferrocenylhexanethiol; V.P., *Vibrio parahaemolyticus*; DA, dopamine; PSA, prostate specific antigen; MIP, molecularly imprinted polymer; HRP, horseradish peroxidase; rHuEPO, recombinant human erythropoietin; ALP, alkaline phosphatase; APBA, 3-aminophenylboronic acid; PBNPs, Prussian blue nanoparticles; OVA, ovalbumin; BA, boronic acid; CPS, carboxy-functionalized polystyrenespheres; PANI, polyaniline; CuNPs, copper nanoparticles; LCA, *Lens culinaris* agglutinin; AgNPs, silver nanoparticles; AFP, alpha fetoprotein.

## 3. Boronate-Affinity-Based Fluorescent Assays and Imaging

As a consequence of their high simplicity and sensitivity, fluorescence biosensors have become important tools for the detection of different biological species. By modifying the fluorescent molecules or nanomaterials with boronic acids, the binding event between boronic acid and *cis*-diol-containing substance can be converted to the signal change of fluorescence intensity or emission band [139,140,141,142,143,144,145]. According to the principle of signal change, boronic acid-based fluorescent methods can be mainly divided into three categories: binding-induced fluorescent enhancement, aggregation-induced quenching or emission, and sandwich boronate-affinity biosensors (Table 2). In addition, boronic acid-based fluorescent hydrogelators have been elaborately synthesized to prepare *cis*-diol-responsive hydrogel-based soft sensors for fluorescent sensing and controlled drug release [66,146,147]. Fluorescence boronic acid derivatives have been combined with non-invasive techniques to design novel devices for continuous physiological monitoring and bioimaging of *cis*-diol-containing species because of their advantages of high-resolution and real-time detection [148]. Notably, Geddes’s group developed a daily and disposable glucose-sensing contact lens based on boronic acid-modified fluorophores for continuous ophthalmic sensing of glucose [149,150,151]. 

### 3.1. Binding-Induced Fluorescent Enhancement

**Figure 7:**
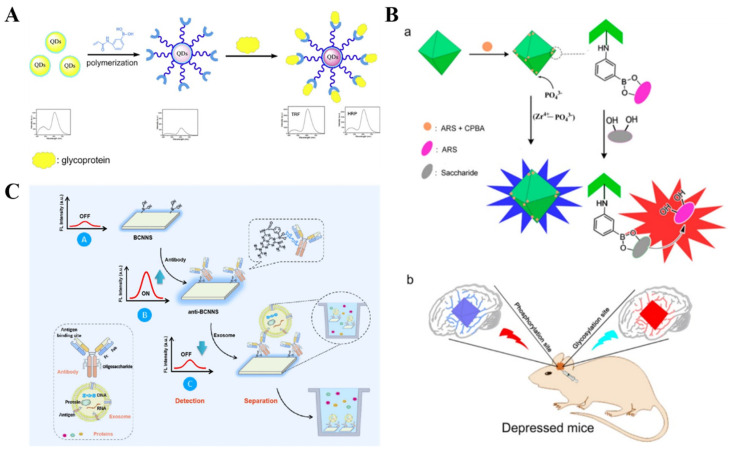
(**A**) Schematic illustration of the fluorescence “turning on” HRP and TRF by using boronic acid-modified QDs [157]. Copyright 2017, Elsevier. (**B**) Schematic illustration of (**a**) proposed mechanism of the fluorescence probe for detection of the levels of glycosylation and phosphorylation based on Zr(IV)-MOF nanomaterials and (**b**) in situ fluorescence imaging of the levels of glycosylation and phosphorylation in depressed mice. Copyright 2020, American Chemical Society. (**C**) Schematic illustration of the separation-detection integrated fluorescent immunoassay for the integrated exosome profiling based on BCNNS [162]. Copyright 2021, American Chemical Society.

Boronic acid is an electron-withdrawing group that can quench the fluorescence of the adjacent fluorophore through a photo-induced electron transfer (PET) process [152,153,154,155]. The covalent interaction between boronic acid and a *cis*-diol-containing substance can block the electron transfer and restore the quenched fluorescence. In the “turn-off-on” detection mode, the fluorescence of nanomaterials modified with boronic acid molecules wasquenched due to the PET process from nanomaterials to the boron moieties [156]. Based on this fact, Chang et al. reported “turn-on” fluorescence biosensors for the detection of HRP and transferrin (TRF) using boronic acid-functionalized quantum dots (QDs) (Figure 7A) [157]. In this study, Mn-doped ZnS QDs were coated with boronic acid-functionalized polymers via a one-step sol-gel polymerization technique. Boronic acid groups quenched the fluorescence of QDs. HRP or TRF could react with boronic acid groups to form boronate ester bonds, thus leading to the recovery of fluorescence. In addition, Zhang et al. prepared a MOF-based fluorescent nanoprobe for the detection and imaging of phosphorylation and glycosylation based on the Zr(IV)-phosphate and boronate-affinity interactions, respectively (Figure 7B) [158]. In this work, 3-carboxybenzeneboronic acid was reacted with alizarin red to form a complex that could be embedded in UIO-66-NH_2_. The glycosyl site then reacted with the complex to release alizarin red, leading to the fluorescence recovery. Meanwhile, phosphorylated sites could interact with the metal node Zr(IV) of UIO-66-NH_2_ to interrupt the metal-ligand charge transfer, also restoring the fluorescence. To lower the pKa value and stabilize the boronic esters in neutral pH media, Wulff-type boronic acid derivatives consisting of an amine adjacent to the boric group were employed to modify the fluorescent nanomaterials [159]. g-C_3_N_4_ nanosheets have intrinsic N atoms in both the backbone and edges. Boronic acid-modified g-C_3_N_4_ (B-g-CN) nanosheets exhibit the similar features of Wulff-type boronic acid derivatives [160]. Wang et al. demonstrated that boronic acid groups could quench the fluorescence of g-C_3_N_4_ nanosheets through the PET process. The interaction of glycoprotein immunoglobulin G (IgG) and boronic acid caused the fluorescence recovery [161].

On the contrary, the covalent interaction between boronic acid and glycan can cause the fluorescence of nanomaterials to decrease, while the presence of some substances can recover the quenched fluorescence, achieving “turn off-on-off” detection. For example, Wang et al. reported a separation-detection integrated fluorescent immunosensor for profiling exosomes using Wulff-type B-g-CN nanosheets (BCNNS) [162]. As displayed in Figure 7C, modification of g-C_3_N_4_ with boronic acids decreased the fluorescence. The BCNNS could capture the glycoproteins (antibodies) under physiological conditions through the formation of boronate ester bonds, resulting in fluorescence enhancement. After the capture of exosomes by antibodies, the exosome-loaded BCNNS were separated by slight centrifugation and re-dispersed in buffer. Furthermore, the fluorescence signal of BCNNS at 440 nm decreased again, realizing the integration of separation and detection without damaging exosomes during ultracentrifugation.

### 3.2. Aggregation-Induced Quenching or Emission

**Figure 8:**
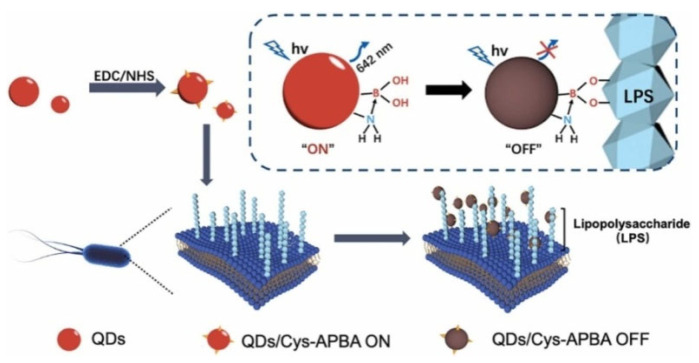
Schematic illustration of the Wulff-type QDs/Cys-APBA for bacteria detection and its fluorescent response to the interaction with the LPS of the outer surface cell wall of *Escherichia coli* [167]. Copyright 2022, Elsevier.

Most fluorescent organic dyes and nanomaterials suffer from an intrinsic shortcoming of aggregation-induced quenching (ACQ) at high concentrations or in the aggregate or solid state. The *cis*-diol-containing biological-related substances can bind with plenty of boronic acid-modified fluorescent probes, subsequently leading to the ACQ effect [163]. For example, glucose can induce the aggregation of boronic acid-modified carbon dots (CDs), leading to fluorescence quenching [164,165,166]. Ye et al. reported an aggregated-induced fluorescence “turn-off” bioassay of Gram-negative bacteria using Wulff-type boronic acid-modified QDs [167]. As shown in Figure 8, Wulff-type L-cysteine (Cys)-APBA consisting of an amine adjacent to the boronic acid group was used to modify QDs (QDs/Cys-APBA), lowering the pKa value and stabilizing the boronic ester in neutral pH medium. QDs/Cys-APBA could interact with LPS on the outer surface of bacteria (*Escherichia coli* and *P. aeruginosa*) over a wide range of pH from 5.0 to 9.0. The aggregation of QDs resulted in fluorescence quenching. Zhang et al. suggested that glycoprotein HRP could induce the aggregation and fluorescence quenching of boronic acid-decorated carbon nanodots (CNDs) [168]. However, the “turn-off” detection system may interfere with other substances in the complicated matrix, leading to a “false positive” result.

**Figure 9:**
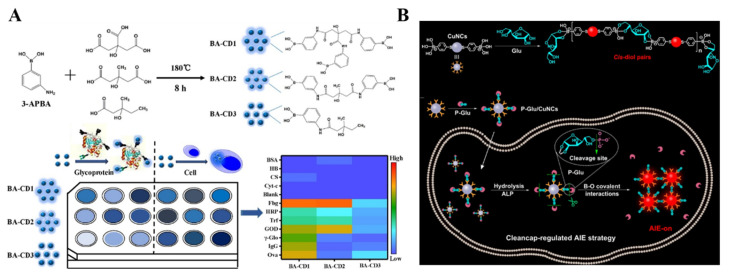
(**A**) Schematic illustration of one-step synthesis strategy for fabrication of BA-CDs and fluorescence sensor array based on three BA-CDs for identification of glycoproteins and cells [172]. Copyright 2021, Elsevier. (**B**) Schematic illustration of the proposed mechanism for specific recognition between MPBA-stabilized CuNCs and glucose and the cleancap-regulated AIE strategy for imaging of ALP activity [173]. Copyright 2020, American Chemical Society.

Since Tang’s group proposed the concept of aggregation-induced emission (AIE) in 2001, various molecules and nanomaterials with AIE properties have been intensively used to develop boronate-affinity-based fluorescent biosensors [169,170]. For example, Zhang et al. prepared boron-doped graphene QDs for glucose determination based on the AIE effect [171]. Chen et al. developed boronic acid-containing CDs (BA-CDs) arrays for sensitive identification of glycoproteins and cancer cells [172]. As illustrated in Figure 9A, three types of BA-CDs were prepared with APBA as the boron doping source and differentorganic acids as the variant precursors. The BA-CDs could bind to the *cis*-diols of glycoproteins and form rigid structure aggregates, leading to the restriction of intramolecular rotation and enhanced emission. The three BA-CDs exhibited distinct fluorescence responses toward glycoproteins due to the difference in glycosylation content, molecular weight, and other chemical compositions of glycoproteins. Thus, glycoproteins and cancer cells with different glycoprotein compositions could be selectively identified. In addition, the controlled assembly of AIE systems based on specific enzyme-substrate recognition and *cis*-diol/boronic acid conjugation can obviously enhance the fidelity and selectivity of sensing systems. Huang et al. designed a cleancap-regulated copper nanoclusters (CuNCs)-based AIE strategy for specific detection of ALP [173]. As illustrated in Figure 9B, CuNCs with AIE property were prepared with MPBA as both reducing agent and stabilizing ligand. Glucose could induce the aggregation of CuNCs via the interactions of boronic acid groups on CuNCs and two *cis*-diols in glucose, leading to the red AIE emission. CuNCs were modified with D-glucose 6-phosphate (P-Glu) as the capper and substrate. ALP induced the cleavage of the phosphate group, and the exposed free 5,6-diol of glucose could react with boronic acid groups on other CuNCs, resulting in the red AIE luminescence. This method with dual-recognition properties (ALP/P-Glu and 5,6-diol/MPBA) was applied for in situ imaging of ALP activity in cells.

**Figure 10:**
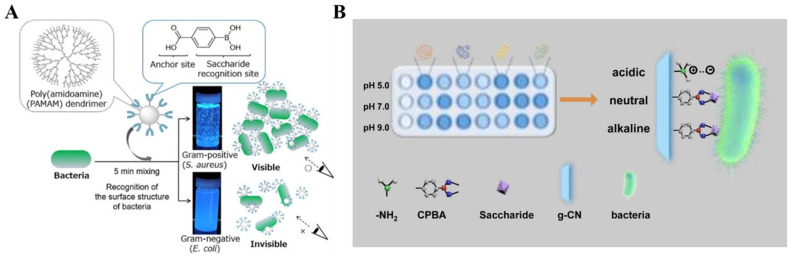
(**A**) Schematic illustration of the discrimination of Gram-positive and Gram-negative bacteria by B-PAMAM(G4) [176]. Copyright 2019, American Chemical Society. (**B**) Schematic illustration of the sensing array based on the (+)BA-g-CN nanosheets for the discrimination of pathogenic bacteria [179]. Copyright 2021, Elsevier.

Due to the multiple boric moieties on the nanoparticle surface, boronic acid-modified NPs have multiplebinding recognition sites for *cis*-diol-containing targets [174]. The surface of bacterial cells has different types of abundant saccharides (e.g., peptidoglycan and lipopolysaccharide). Thus, different bacteria have distinct binding affinity toward boronic acid derivatives at various conditions [175]. Tsuchido et al. reported the rapid discrimination of Gram-positive and Gram-negative bacteria using boronic acid-modified poly(amidoamine) generation 4 (B-PAMAM(G4)) (Figure 10A) [176]. In this study, B-PAMAM(G4) interacted with bacteria to form aggregates, leading to decreased turbidity. The size of the aggregates was dependent on the type of bacteria and the solution pH. In basic pH (9.0), both Gram-positive and Gram-negative bacteria promoted the formation of visible aggregates, but at neutral pH, only Gram-positive bacteria allowed the formation of visible aggregates. The pH-dependence is involved in the difference in boronate affinity between bacterial surface saccharide and the boronic acid moiety of B-PAMAM(G4) dendrimer. They further investigated the mechanism of bacterial recognition by boronic acid-modified dendrimers with different surface properties and found that boronic acid-based nanoprobes can be engineered to exhibit diverse selectivity toward strains, species, or a certain group of bacteria [177,178]. In addition, Yang et al. constructed a sensing array for discriminating pathogenic bacteria based on the distinctive properties of Wulff-type boronic acid-decorated g-C_3_N_4_nanosheets ((+)BA-g-CN) at various pH values [179]. As shown in Figure 10B, bacterial cells could interact with (+)BA-g-CN through either electrostatic or boronate interactions at various pH values. Furthermore, the sedimentation of the bacteria-(+)BA-g-CN composites resulted in a decrease in the fluorescence intensity of the supernatant.

### 3.3. Sandwich Fluorescence Assays

**Figure 11:**
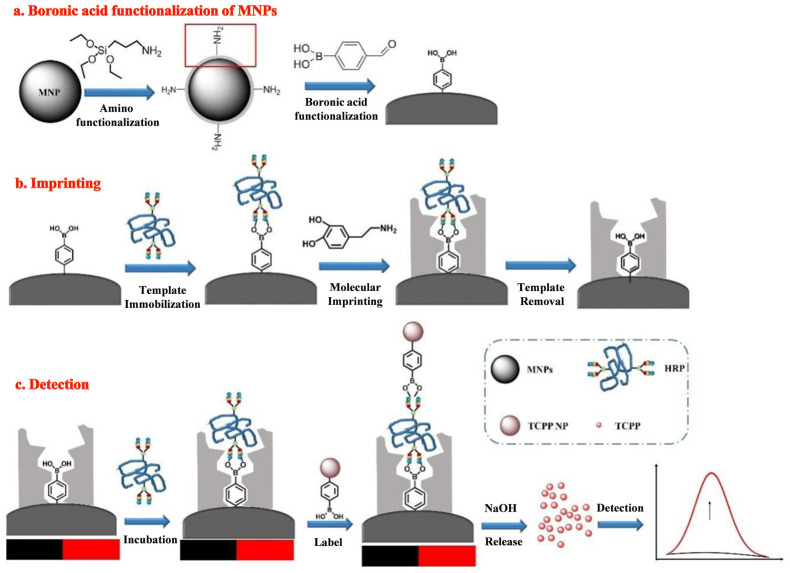
Schematic illustration of the preparation of fluorescence “turn on” detection of HRP based on boranate affinity sandwich assay and nanoparticle signal amplification. (**a**) BA modification MNPs; (**b**) HRP imprinting on the surface of BA-MNPs using surface oriented imprinting method, and (**c**) detection of HRP based on boranate affinity sandwich assay and TCPP NPs signal amplification [183]. Copyright 2020, Elsevier.

Biosensors based on the simple boronic acid molecule show relatively poor selectivity toward *cis*-diol substances due to the interference effect ofother *cis*-diols. Thus, sandwich boronate-affinity biosensors are attractive for selectively detecting *cis*-diol targets [180,181]. Recently, boronate-affinity-based surface MIPs have been used to develop sandwich fluorescence assays of glycoproteins [182]. For example, Lu et al. developed a fluorescence “turn-on” MIP biosensor for the detection of glycoprotein HRP [183]. As shown in Figure 11, an ultra-thin boronate-affinity-based MIP film was formed on the surface of magnetic NPs (MNPs). The self-assembled tetra(4-carboxyphenyl) porphyrin (TCPP) NPs were modified with APBA (BA-TCPP NPs) to act as the signal tags. HRP captured by the imprinted MNPs was recognized by BA-TCPP NPs. After magnetic separation, the adsorbed TCPP NPs led to the release of a large number of TCPP molecules under treatment with an alkaline solution, generating an amplified fluorescence signal. 

**Figure 12:**
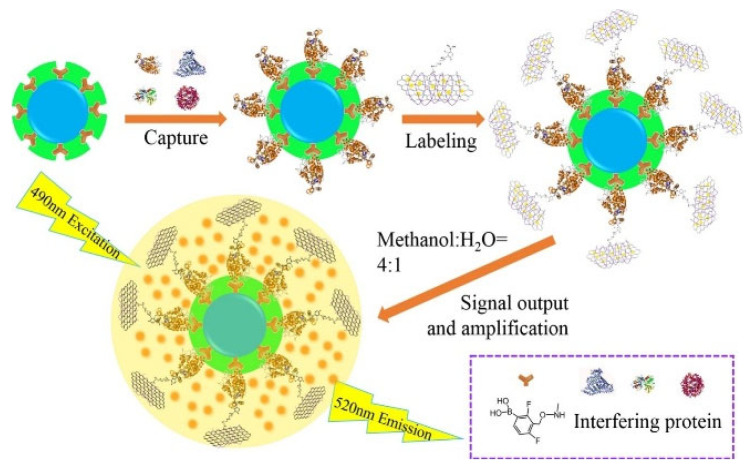
Schematic illustration of a MIPs-coated MNPs-based biosensor for detection of glycoproteins using BA-modified FITC-loaded GO as signal labels [185]. Copyright 2021, Elsevier.

Nanomaterials with a large surface area can be used as carriers to load fluorescent dyes as signal labels for sandwich assays. Yu et al. reported a MIP-coated MNP-based biosensor for the detection of glycoproteins using boronic acid-modified carbon nanotubes to load FITC molecules [184]. Bai et al. developed a fluorescent sandwich biosensor for the detection of HRP and CEA [185]. As shown in Figure 12, boronic acid-modified FITC-loaded GO nanocomposites were used as the labels for signal amplification. FITC molecules were released by the mixture of methanol and H_2_O, producing a strong fluorescence signal. The proposed sandwich biosensor showed a detection limit of 23 fg/mL or 1.2 fg/mL for HRP or CEA, respectively.

**Table 2:** Analytical performances of boronic acid-based fluorescence biosensorsfor the detection of glycoproteins, bacteria, andexosomes.

Signal Probes	Target	Linear Range	LOD	Ref.
APBA-CuNCs	Ovalbumin	5–220 nM	2.6 nM	[141]
UiO-66-NH_2_@B(OH)_2_	SA	0.05–2.5 mM	0.025 mM	[142]
MPBA-QDs	TRF	0.1–10 μM	5.69 nM	[156]
BA-QDs	HRP and TRF	0.3–0.7 μM and 0.4–0.9 μM	0.144 nM and 0.336 nM	[157]
BA-g-C_3_N_4_	IgG	6.7–67 nM	2.2 nM	[161]
BA-g-C_3_N_4_	Exosome	8 × 10^3^–1 × 10^5^ particles/mL	2484 particles/mL	[162]
APBA-QDs	*Escherichia coli* and *P. aeruginosa*	1.12 × 10^3^–1.12 × 10^8^ CFU/mL	58 and 97 CFU/mL	[167]
BA-CNDs	HRP	3.3–333.3 μg/mL	0.52 μg/mL	[168]
P-Glu/CuNCs	ALP	0.56–30 U/L	0.17 U/L	[173]
BA-QDs	HCG	0.24–62.5 mIU/mL	0.19 mIU/mL	[181]
BA-TCPPs	HRP	0.1 μg/L–10 mg/L	0.042 μg/L	[183]
BA-FITC-CNTs	HER2	31 fg/mL–5 μg/mL	14 fg/mL	[184]
BA-FITC-GO	HRP and CEA	1 × 10^−7^ pg/mL–5 μg/mL and 2.4 × 10^−6^–10 ng/mL	23 and 1.2 fg/mL	[185]

Abbreviation: BA, boronic acid; g-C_3_N_4_, graphitic carbon nitride; IgG, immunoglobulin G; SA, sialic acid; APBA, 3-aminophenylboronic acid; CuNCs, copper nanoclusters; QDs, quantum dots; HRP, horseradish peroxidase; TRF, transferrin; HCG, human chorionic gonadotropin; MPBA, 4-mercaptophenylboronic acid; CNDs, carbon nanodots; HRP, horseradish peroxidase; P-Glu, D-glucose 6-phosphate; FITC, fluorescein isothiocyanate; GO, graphene oxide; CNTs, carbon nanotubes; HER2, human epidermal growth factor receptor-2; TCPPs, tetra(4-carboxyphenyl) porphyrin.

**Figure 13:**
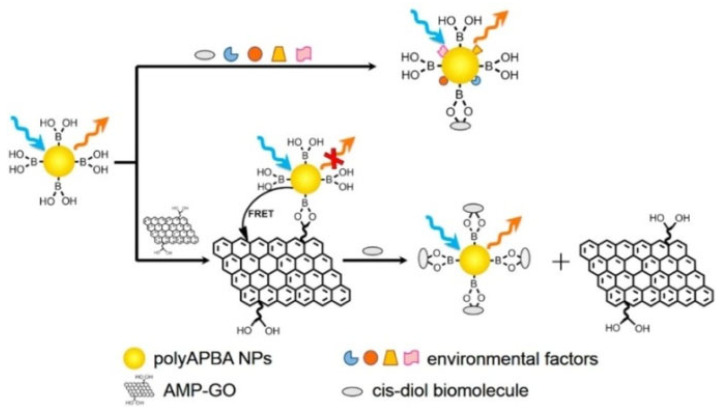
Schematic illustration of a FRET scheme based on poly(mAPBA) NPs and AMP-GO for the selective sensing of *cis*-diol biomolecules [189]. Copyright 2016, American Chemical Society.

The distance (typically in the range of 2–10 nm) between the donor-acceptor pair can significantly influence the efficiency of Förster resonance energy transfer (FRET). The specific interaction between boronic acid derivatives and *cis*-diol biomolecules can modulate the distance, leading to a change in fluorescence intensity. Since the traditional dye-dye FRET pairs are always susceptible to environmental factors, various nanomaterials with different optical properties have been explored as FRET donors or acceptors [186,187,188]. Wang et al. reported a FRET biosensor for the detection of *cis*-diol biomolecules using poly(m-aminophenylboronic acid) nanoparticles (poly-(mAPBA) NPs) and 5′-monophosphate modified GO (AMP-GO) as the donor and acceptor, respectively [189]. As illustrated in Figure 13, poly-(mAPBA) NPs with stable fluorescence properties and appropriate resistance to environmental factors were conjugated with AMP-GO via the interactions between boronic acid moieties and the *cis*-diol groups. The fluorescence of poly-(mAPBA) NPs was quenched by AMP-GO through the FRET process. However, glucose and transferrin could competitively interact with poly-(mAPBA) NPs, inhibiting the FRET process and recovering the fluorescence. In addition, Chang et al. reported the fluorescent bioassay of glycoprotein based on the FRET between QDs and AuNPs [190]. In this study, glycoprotein decomposed the interaction between the boronic acid moiety on AuNPs and glucosamine on the surface of QDs, leading to fluorescence recovery.

### 3.4. Fluorescent Imaging

**Figure 14:**
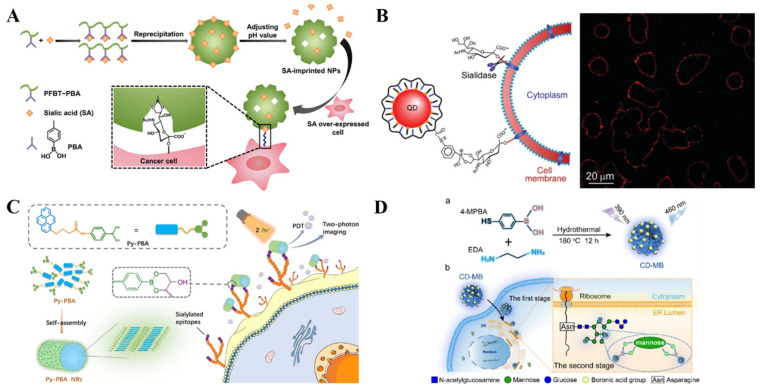
(**A**) Schematic illustration of the preparation of sialic acid (SA)-imprinted CPNPs and the mechanism of their selectivity toward cancer cells [193]. Copyright 2017, American Chemical Society. (**B**) Schematic illustration of Py-PBA NRs for two-photon imaging of cell surface SAs and PDT [194]. Copyright 2017, American Chemical Society. (**C**) Schematic illustration of QD conjugation and the use of QD probes for specific labeling of SA on living cells [195]. Copyright 2011, American Chemical Society. (**D**) Schematic illustration of (**a**) the synthetic procedure for CD-MB with BA groups on the surface and (**b**) its ER-targeting ability via a two-stage cascade ER recognition process in living cells [196]. Copyright 2022, American Chemical Society.

Effective techniques and methods for in situ analysis of glycans on living cell surfaces are of great importance for clinical diagnostics and therapeutics. The abnormal expression of sialic acid is closely related to various diseases such as cardiovascular diseases, neurological diseases, and cancers. Fluorescein isothiocyanate (FITC)-doped phenylboronic acid (PBA)-modified SiO_2_ NPs and PBA-tagged polydiacetylene-liposomes were employed for the in situ imaging of sialic acid-terminated glycans on living cell surfaces, respectively [191,192]. To improve the selectivity toward sialic acid and cancer cells, Liu et al. reported the imaging of target cancer cells by integration of boronate-affinity MIPs with fluorescent conjugated polymer nanoparticles (CPNPs) [193]. As presented in Figure 14A, the sialic acid-imprinted CPNPs prepared by fluorescent conjugated polymers with PBA side chains showed a stronger fluorescence than the unmodified nanoparticles. The imprinted NPs could selectively bind to the sialic acid-overexpressed cancer cells, successfully discriminating between the two types of cells with and without sialic acid groups. However, such a one-photon excited fluorescence imaging method involves the excitation of ultraviolet-visible light, which may be harmful to cells. To minimize the cell damage and improve the imaging performance, Li et al. reported in-situ two-photon imaging of cells and photodynamic therapy (PDT) using self-assembled nanorods of PBA-functionalized pyrene (Py-PBA) [194]. As illustrated in Figure 14B, Py-PBA with a hydrophilic tail and a hydrophobic head was used as the building block to prepare hydrophilic nanorods (Py-PBA NRs) through the self-assembly technique by the π-π stacking interaction and hydrophobic effect. The self-assembled Py-PBA NRs showed high specificity toward the sialic acid by the multivalent interaction, realizing the in-situ two-photonimaging of sialic acid on the living cell surface. Moreover, under two-photon irradiation, the bound Py-PBA NRs could produce ^1^O_2_ to cause the necrosis of cells for PDT.

Nanomaterials with outstanding optical properties can also be used in fluorescent bioimaging. Liu et al. prepared QDs with PBA tags for specific and efficient labeling of sialic acid on living cells (Figure 14C) [195]. The PBA-functionalized QDs could label and continuously track the sialic acid moieties on the cell surface in one step without any pretreatment. The QDs-labeled sialic acids were quickly internalized via endocytosis and eventually distributed in the perinuclear region. However, the inherent biotoxicity and the time-consuming multiple steps greatly limit the application of QDs for in vivo imaging of cancer cells. Recently, Wu et al. used fluorescent carbon dots (CDs) with boronic acid groups for dynamic visualization of endoplasmic reticulum (ER) stress in living cells [196]. As shown in Figure 14D, the fluorescent CD with a boronic acid group (CD-MB) was prepared with 4-MPBA and ethylenediamine as the precursors through a facile one-pot hydrothermal method without additional modification. The positively charged, lipophilic CD-MB could rapidly cross multiple membrane barriers and accumulate in the ER. Next, it specifically labeled the ER via the interaction between boronic acid and the o-dihydroxy group of mannoses in the ER lumen. The exterior stimuli-induced ER stress could be visualized by monitoring the change in fluorescence intensity and distribution of CD-MB due to the dynamic transfer of mannose induced by autophagy.

## 4. SERS Biosensors

**Figure 15:**
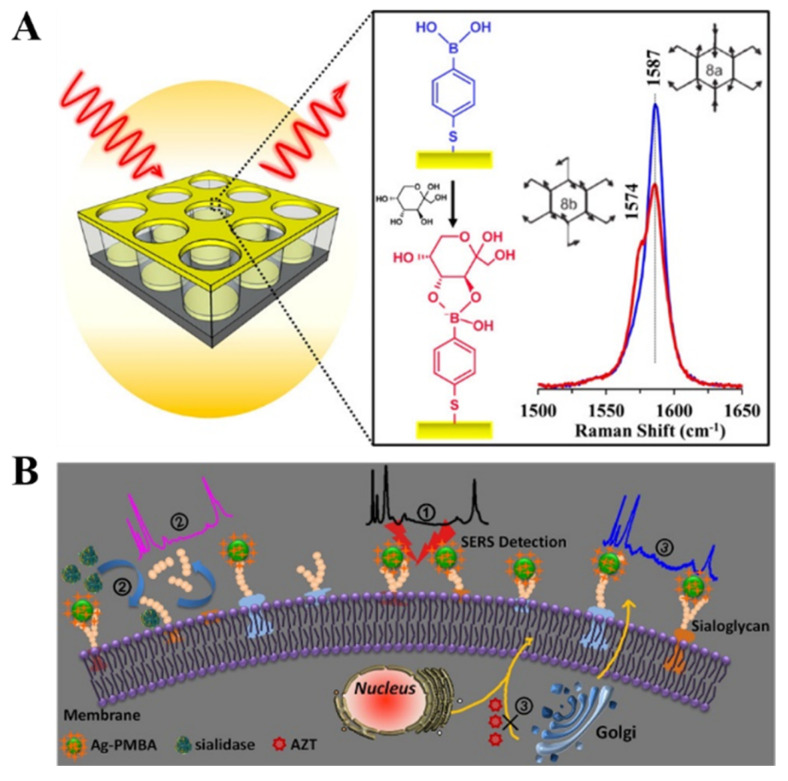
(**A**) Schematic illustration of MPBA on a quasi-three-dimensional plasmonic nanostructure array for sensitive and fast detection of fructose using SERS [200]. Copyright 2014, American Chemical Society. (**B**) Schematic illustration of the specific study of dynamic expression of sialoglycans by SERS spectroscopy with a nanosensor in molecular recognition [202]. Copyright 2017, Elsevier.

SERS spectroscopy can offer fingerprint vibrational information of surface species, which exhibits ultrahigh sensitivity due to electromagnetic and chemical enhancement. Such a technique shows the remarkable advantages of less susceptibility to sample environment, rapid readout speed, and possibility for on-site and non-invasive measurement within an acceptable range ofsafety and patient tolerability [197,198]. Therefore, SERS spectroscopy has been widely used in the field of chemical and biological analysis. Label-free SERS detection of *cis*-diol-containing species can be realized by collecting the Raman signal from targets. However, sensitivity and selectivity are always poor due to the inherently small Raman scattering cross section of analytes. Furthermore, the complex chemical structures of proteins make the spectra complicated and the identification difficult. It is an effective strategy to modify the SERS-active substrate with the self-assembled monolayer (SAM) showing Raman-active and recognition-active abilities, which can act as the receptor and reporter to simultaneously capture the target and provide a Raman signal for SERS measurement [199]. For example, Sun et al. reported a SERS method for fructose detection using MPBA-modified gold surfaces [200]. As illustrated in Figure 15A, a quasi-three-dimensional plasmonic nanostructure array was modified with an MPBA SAM via the thiol-gold interaction. In addition to target recognition, the benzene ring of MPBA could amplify the SERS signal with extra chemical enhancement, leading to the shielding of the background noise of complex media. The binding between fructose and boronic acid could result in the symmetric breaking of MPBA and the change of area ratio between totally symmetric 8a ring mode and non-totally symmetric 8b ring mode, achieving the sensitive detection of fructose.

Generally, SERS nanotags are composed of plasmonic metallic NPs and organic Raman reporter molecules. The characteristic Raman signal from nanotags can be changedafter interaction with targets [201]. For example, Liang et al. reported a SERS nanosensor for in-situ monitoring the levels of sialoglycan and its dynamic expression process in different types of cellsby using SERS nanotag MPBA-AgNPs (Figure 15B) [202]. In this work, the interaction between MPBA and sialogycan resulted in a change in the molecular vibrational mode of MPBA, which could be readily monitored by SERS. However, the complex composition of cells may cause matrix interference in the SERS fingerprint region. SERS reporters with chemical groups (e.g., alkyne, cyano, and azide) can produce a distinct and strong Raman band in the cellular silent region. For this consideration, He et al. prepared a background-free SERS probe with a cyano group for the detection of sialic acid on the cell surface [203].

**Figure 16:**
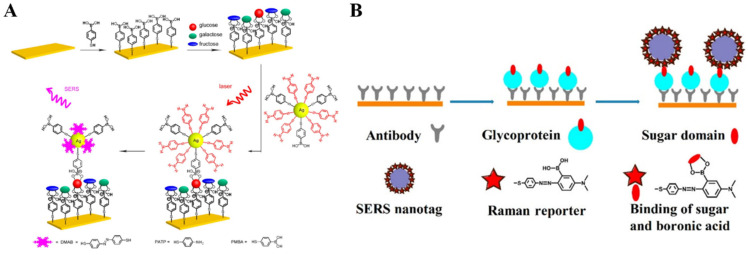
(**A**) Schematic illustration of a glucose sandwich assay using a PMBA-modified self-assembled monolayer on a smooth gold-coated silicon wafer and SERS tags of AgNPs modified with PATP and PMBA [205]. Copyright 2015, American Chemical Society. (**B**) Schematic illustration of an immunosensor for determination of glycoprotein based on SERS nanotags DTDPA-DMAPBA-modified AuSPs [207]. Copyright 2017, American Chemical Society.

Unlike label-free SERS detection, sandwich assays with receptor-modified substrates or SERS nanotags exhibit high sensitivity and selectivity [204]. Bi et al. reported a sandwich method for glucose detection based on in situ-generated Raman reporters [205]. As presented in Figure 16A, a smooth gold-coated slide was coated with a SAM of MPBA for glucose capture. AgNPs were modified with both MPBA and p-aminothiophenol (PATP) as SERS nanotags. After the formation of sandwich complex, PATP molecules were in-situ converted into the Raman reporter of 4,4′-dimercaptoazo-benzene (DMAB) under 785 nm laser irradiation during the measurement. Moreover, the SERS technique can be combined with the traditional ELISA method for the construction of efficient and sensitive biosensors with SERS nanotags [206]. Typically, Ma et al. reported an immunosensor for the determination of glycoprotein based on azobenzene derivative-modified gold superparticles (AuSPs) (Figure 16B) [207]. AuSPs were prepared with APBA as the reducing agent, and the oxidation product of poly(3-aminophenylboronic acid) (PAPBA) was used as the capping agent. The PAPBA-capped AuNPs could self-assemble into AuSPs through the π-π stacking of PAPBA. AuSPs were further functionalized with bis [4,4′-(dithiodiphenylazo)-1-(N,N′-dimethylamino)-3-phenylboronic acid] (DTDPA-DMAPBA). The Raman signal of DTDPA-DMAPBA was greatly enhanced by the high-density hot spots on AuSPs due to the sharp tips and nanogaps.

**Figure 17:**
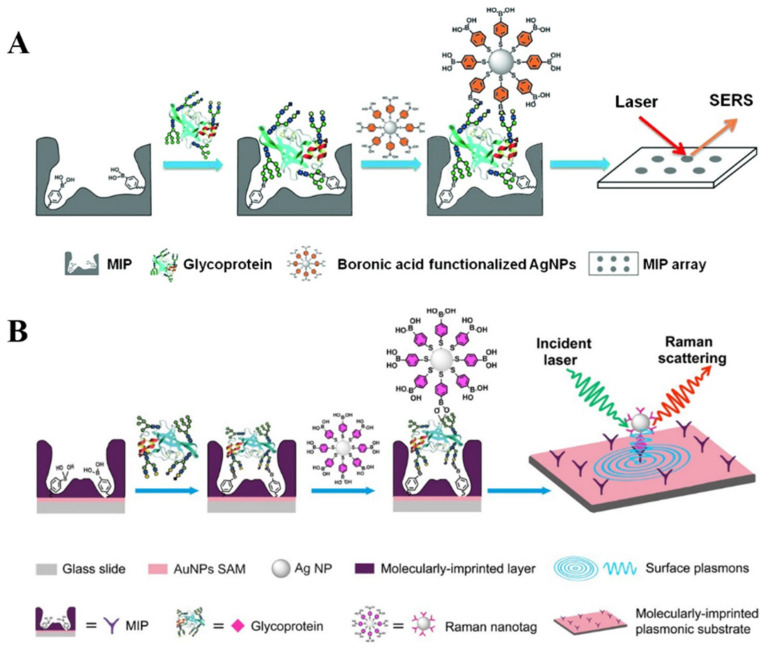
(**A**) Schematic illustration of the boronate-affinity sandwich assay of glycoproteins [210]. Copyright 2014, Wiley-VCH. (**B**) A schematic illustration of the MIP-based plasmonic immunosandwich assay for the detection of target glycoproteins [211]. Copyright 2017, American Chemical Society.

The combination of MIPs with boronic acid-modified SERS substrates or nanotags without the use of biometric components is an attractive approach for the design of biosensors [208,209]. Ye et al. developed a boronate-affinity sandwich method for the detection of glycoproteins in complex samples [210]. As shown in Figure 17A, the macroporous boronate-affinity MIPs were used as the artificial receptors to specifically capture glycoproteins. After other species were washed away, boronic acid-modified AgNPs were added to label the captured targets and act as Raman reporters to provide a strong signal. To further improve the sensitivity, Muhammad et al. reported a sandwich plasmonic immunoassay for glycoprotein detection using boronate-affinity MIPs with plasmon-enhanced Raman scattering (Figure 17B) [211]. In this work, the glass slide substrate was decorated with the SAM of AuNPs and then modified with boronate-affinity MIPs. The plasmonic gold SAM greatly enhanced the Raman signals of AgNPs-based SERS nanotags.

**Figure 18:**
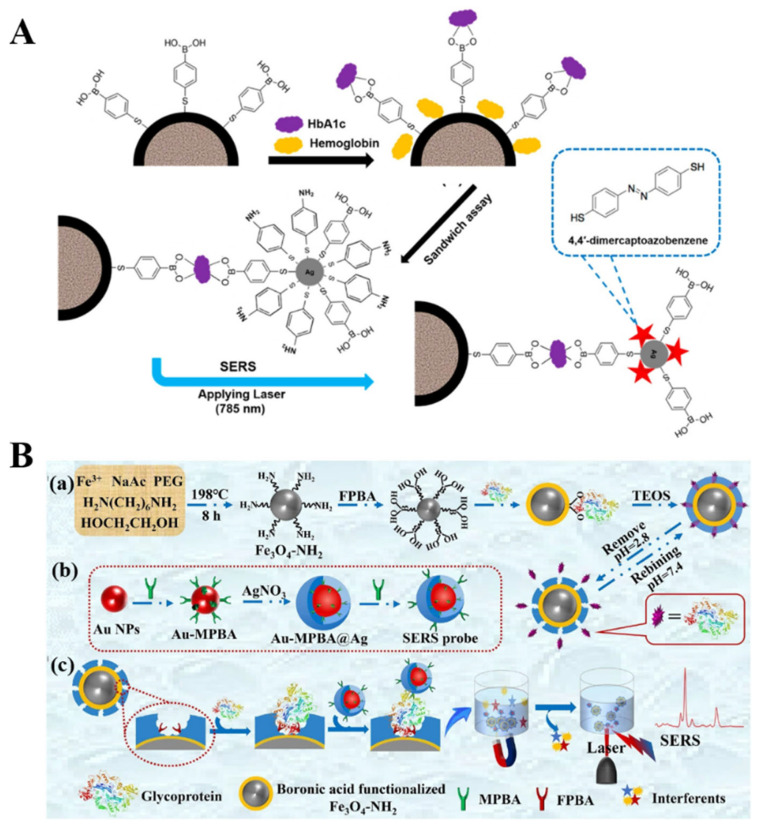
(**A**) Schematic illustration of a boronate-affinity-based sandwich assay for determination of glycated hemoglobin by using an MPBA-modified SERS nanotag [214]. Copyright 2016, American Chemical Society. (**B**) Schematic illustration of (**a**) the preparation procedure of boronate-affinity MMIPs, (**b**) the preparation procedure of boronic acid functionalized SERS probes, and (**c**) the schematic of sandwich assay construction and detection of target glycoproteins [215]. Copyright 2022, Elsevier.

Magnetic micro/nanocomposites have been widely used as effective substrates to promote the isolation of targets from complex samples because of their facile separation and function. They can significantly improve the sensitivity, reliability, and anti-interference ability of SERS methods. After the capture and magnetic separation of *cis*-diol-containing substances by receptor-modified magneticmicro/nanocomposites, boronic acid-functionalized SERS nanotags can specifically recognize the targets through the boronate-affinity interactions [212,213]. For example, Usta et al. developed a boronate-affinity-based sandwich assay for the determination of glycated hemoglobin with MPBA-modified SERS nanotags as the reporters [214]. As illustrated in Figure 18A, magnetic polymethacrylate microspheres were coated with Ag shells and further functionalized with MPBA (MPBA-Ag@MagPMMS). After the formation of sandwich complexes and magnetic separation, DMAB was in-situ generated on the surface of PATP and MPBA-modified AgNPs under the illumination of a laser. To enhance the signal intensity of nanotags, Au-reporter@Ag nanotags were synthesized by embedding the Raman reporter molecule between the gold core and silver shell. However, it is prone to being disturbed in complex samples. To overcome this shortcoming, Hu et al. reported a boronite-affinity-based surface-imprinted magnetic platform for the detection of glycoproteins [215]. As shown in Figure 18B, MNPs were coated with the boronate-affinity MIP polymers. After the capture of glycoproteins, MNPs were magnetically separated from the complex matrix. Furthermore, Au-MPBA@Ag nanotags were introduced to identify the captured targets, and the SERS signals were recorded by a portable Raman meter.

## 5. Boronate-Affinity-Based Colorimetric Assays

Owing to their high simplicity, easy operation, and rapid on-site detection, colorimetric assays have proven to be the most convenient methods for quantitative or semi-quantitative analysis. Boronic acid groups can serve as specific binding sites for conjugated dyes. The target recognition by boronic acid can result in achromogenic reaction and achange in absorbance for signal readout that can be monitored by the naked eye or ultraviolet-visible spectrophotometry [216]. Meanwhile, a series of boronic acid-based sensors or arrays have been reported for quantitative sugar analysis, in which the pH change induced by the formation of boronic esters can be measured by pH indicators [217,218,219]. The research progress of boronic acid-based molecular probes for colorimetric detection of small molecules (e.g., glucose, H_2_O_2_, F^−^, and dopamine) has been summarized in several reviews [220,221]. In this section, we primarily focused on colorimetric assays for biomacromolecules using boronic acid-modified molecules and nanomaterials as signal labels.

### 5.1. Boronic Acid-Based Plasmonic Colorimetric Biosensors

**Figure 19:**
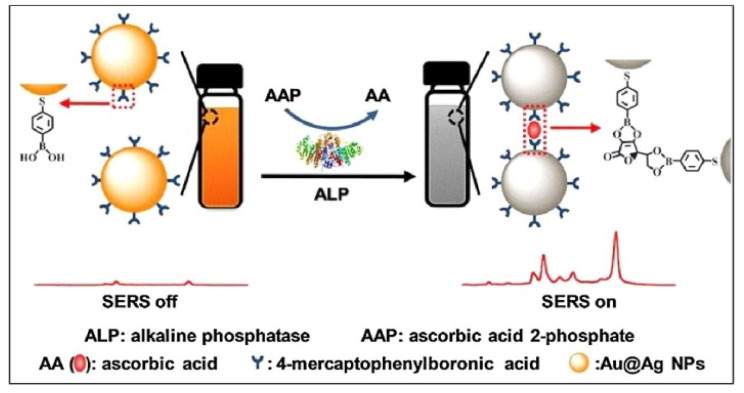
Schematic illustration of a dual-readout strategy for assaying ALP activity based on AA-induced aggregation of MPBA-Au@Ag NPs via its two *cis*-diolinteracting groups to BA moieties on the surface of Au@Ag NPs [231]. Copyright 2017, Elesiver.

The traditional ELISA methods usually require the use of enzymes to catalyze chromogenic reactions for signal readout. However, the enzyme-linked colorimetric assays show poor sensitivity due to the low extinction coefficient of organic chromogens and the interior stability of enzymes. To enhance the sensitivity of colorimetric assays, various metal nanomaterials, including AuNPs, AgNPs, and Au nanorods, have been exploited as substrates to construct plasmonic colorimetric biosensors. The aggregation of AuNPs can result in electromagnetic coupling among the localized surface plasmon resonance (LSPR) of nearby nanoparticles and the color change of solution from red through purple to blue with a red-shifted and broadened LSPR peak [222,223]. Based on the target-responsive property of boronic acid groups, different AuNPs or AgNPs have been utilized in colorimetric bioassays of *cis*-diol-containing species in which boronic acid molecules served as the capping regents or inducers. Boronic acid derivatives with a thiol or amino group can be used to functionalize AuNPs via the gold-thiol interaction or amidation reaction. The resulting boronic acid-modified AuNPs exhibited aspecific *cis*-diol-responsive ability. Targets with multiple *cis*-diol groups can react with boronic acid groups on the surface of AuNPs and induce their aggregation, resulting in the color change [224,225,226,227,228]. For example, it has been demonstrated that glucose and sialic acid could act as a cross-linker to induce the aggregation of APBA-functionalized AuNPs [229,230]. Ascorbic acid (AA) with two pairs of diol groups can interact with boronic acid-modified metal NPs and induce their aggregation. For this view, Zhang reported a colorimetric and SERS dual-mode method for the assay of ALP activity based on AA-induced aggregation of MPBA-modified Ag-coated AuNPs (MPBA-Au@AgNPs) [231]. As presented in Figure 19, ALP promoted the conversion of ascorbic acid 2-phosphate (AAP) into AA. The produced AA could induce the aggregation of MPBA-Au@AgNPs, leading to a change in solution color from bright orange to dark gray. Meanwhile, the formed Raman hotspots in the aggregates greatly amplified the SERS signal.

**Figure 20:**
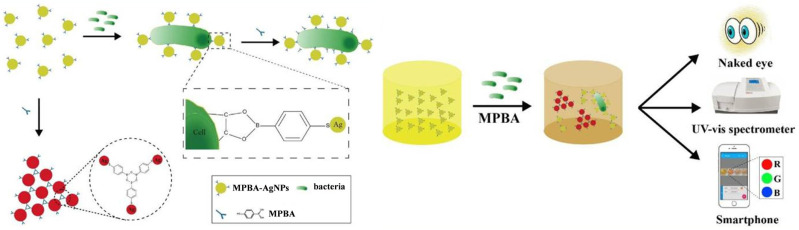
Schematic illustration of (**left**) the mechanism of colorimetric assay of bacteria based on the inhibition of the aggregation of MPBA-AgNPs and (**right**) signal detection methods based on the naked eye, UV-vis spectrometer, and smartphone [238]. Copyright 2018, Elsevier.

On the contrary, boronic acid groups can react with each other to form planar 6-membered boroxine rings via self-dehydration condensation under mild reaction conditions [232]. Thus, MPBA with a thiol groupand a boronic acid moiety can act as an inducer to trigger the aggregation of AuNPs based on the boroxine ring-forming reaction and the gold-thiol interaction [233,234]. The *cis*-diol-containing targets, such as adenosine triphosphate and dihydronicotinamide adenine dinucleotide, can competitively prefer to react with the boronic acid group and inhibit the self-dehydration condensation reaction, leading to the anti-aggregation of AuNPs [235,236]. Bacteria contain rich carbohydrate species on their surfaces that can capture plenty of PBA-functionalized materials [237]. Therefore, Zheng et al. reported a colorimetric bioassay for monitoring bacteria based on the inhibition of MPBA-induced aggregation of AgNPs [238]. As illustrated in Figure 20, Gram-negative bacteria such as *E. coli* could bind with MPBA-functionalized AgNPs via the interactions between the *cis*-diol groups in saccharides on the bacteria cells and the boronic acid groups on AgNPs. This would reduce the number of free AgNPs in solution and thus limit MPBA-induced aggregation of AgNPs. *E. coli* was detected in a dynamic range from 5 × 10^4^ cfu/mL to 1 × 10^7^ cfu/mL, with a detection limit of 0.9 × 10^4^ cfu/mL.

**Figure 21:**
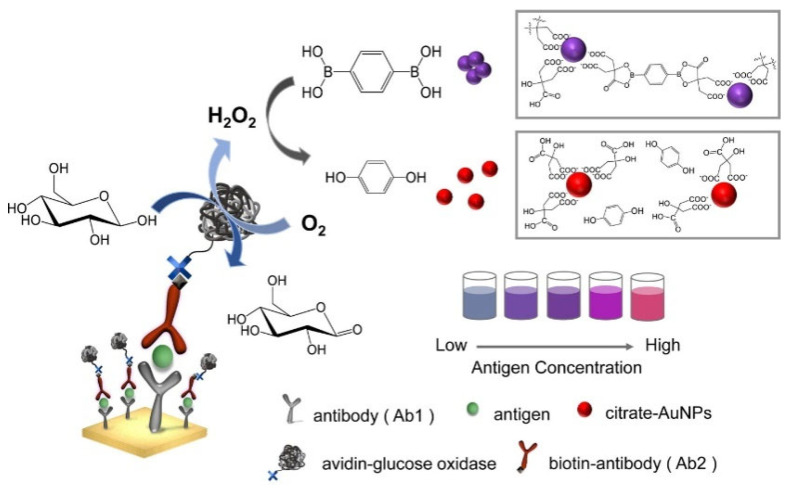
Schematic illustration of Naked-eye readout of plasmonic immunoassays. Detection of target protein via the combination of sandwich immunoassay, avidin-biotin interaction, GOx-mediated oxidation of glucose, H_2_O_2_-induced oxidation of BDBA, and BDBA-triggered aggregation of citrate-capped AuNPs [239]. Copyright 2016, American Chemical Society.

Inducer-mediated aggregation of NPs can also be coupled with an enzymatic reaction for bioassays, in which the concentration or structure of the inducer is modulated by an enzymatic reaction. For example, Yang et al. reported a plasmonic immunoassay for colorimetric detection of rabbit IgG and human PSA based on the H_2_O_2_-inhibited aggregation of citrate-capped AuNPs [239]. As shown in Figure 21, benzene-1,4-diboronic acid (BDBA) could induce the aggregation of citrate-capped AuNPs through the interaction between the *α*-hydroxycarboxylate of citrate and the boronic acidgroup of BDBA [240]. H_2_O_2_ could oxidize the boronic acid into phenol and inhibit the BDBA-induced aggregation of AuNPs. In combination with a glucose oxidase (GOx)-based ELISA immunosensor, the enzymatically generated H_2_O_2_ was proportional to the concentration of the target protein.

### 5.2. Boronic Acid-Based Lateral Flow Immunoassays

**Figure 22:**
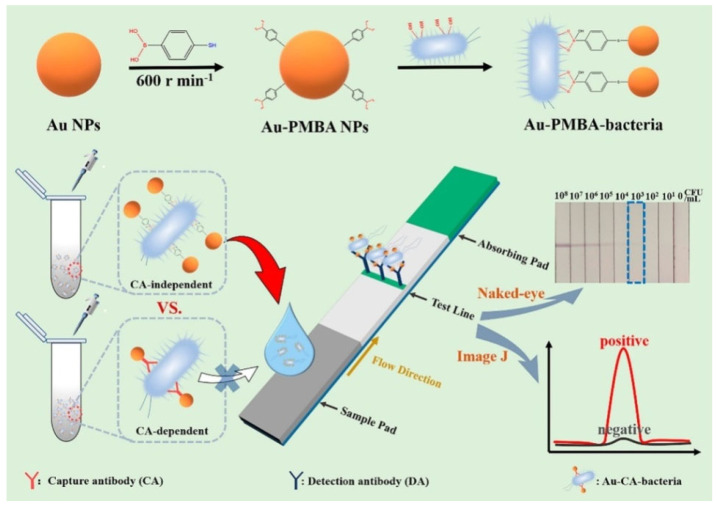
Schematic illustration of the LFIA immunosensor for rapid pathogen detection by using MPBA-AuNPs nanocrabs as a universal bacterial catcher [244]. Copyright 2022, American Chemical Society.

Lateral flow immunoassay (LFIA) platforms have been considered ideal diagnostic tests because of their merits of less time consumption, easy operation, durable stability, and low cost [241,242,243]. Boronic acid-functionalized AuNPs can be used as plasmonic nanoprobes for effective labeling of *cis*-diol-containing targets in LFIA. Wu et al. reported a LFIA platform for the rapid detection of pathogens using MPBA-AuNP nanocrabs as universal bacterial catchers [244]. As illustrated in Figure 22, MPBA-AuNP nanocrabs could recognize and capture Gram-negative and Gram-positive bacteria through covalent binding. The formed MPBA-AuNPs-bacteria complexes were added to the sample pad and then trapped by the antibody-modified test line (T-line), thus generating a red band. This immunosensor exhibited a linear range of 10^3^~10^7^ cfu/mL by taking *Escherichia coli* O157:H7 as an example.

## 6. Others

**Figure 23:**
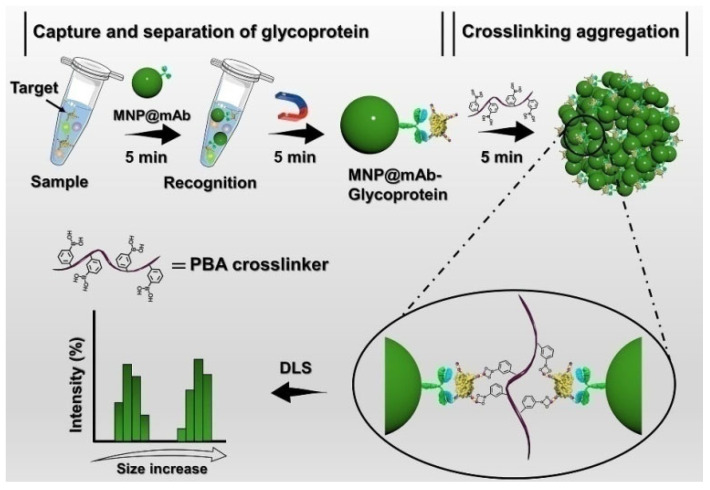
Schematic illustration of the designed boronate-affinity cross linking-amplified DLS immunoassay for glycoprotein detection [251]. Copyright 2022, Wiley-VCH GmbH.

Dynamic light scattering (DLS) is a popularly used technique for characterizing the size distribution of particles by monitoring the variation of scattering light intensity induced by the Brownian motion of particles in solution. Recently, such a technique has been used to develop sensing platforms based on the boronic acid derivative-modulated aggregation/disaggregation of particles [245,246,247,248]. For example, Xiong’s group hasreported boronate-affinity DLS immunoassays for point-of-care (POC) detection of glycoproteins using boronic acid-modified materials as the cross-linking amplifiers [249,250]. One of the examples is the magnetic-assisted DLS immunoassay of glycoprotein in complex samples [251]. As illustrated in Figure 23, antibody-coated magnetic nanoparticles (MNP@mAb) were employed to selectively capture and concentrate the targets. Boronic acid-modified polyethylene glycol was used as the cross-linker to induce the aggregation of the target-captured MNP@mAb by the boronate-affinity interaction, resulting in a significant enhancement in the average hydrodynamic diameter. However, the work did not take into account that antibodies, as glycoproteins, can also bind with boronic acids [95].

**Figure 24:**
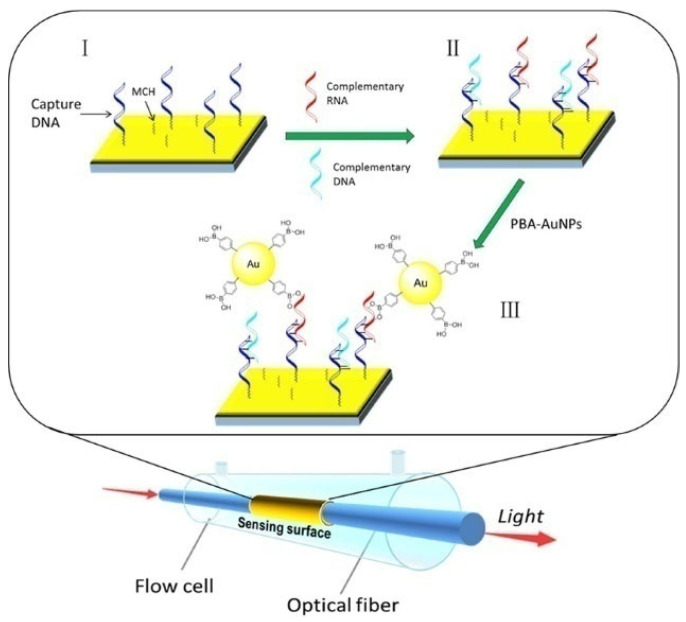
Schematic illustration of miRNA detection by fiber-optic SPR sensing systems: (**I**) Capture DNA modification on the sensing surface. (**II**) Single-stranded RNA or DNA hybrid on the sensing surface. (**III**) PBA-AuNPs selectively bind with RNA to amplify the signal [253]. Copyright 2018, American Chemical Society.

Due to the collective oscillation of electrons at the interface of metal/dielectric, surface plasmon resonance (SPR) can sensitively monitor the refractive index change around the metal surface. Boronic acid-modified AuNPs have been used as the signal labels to improve the sensitivity due to the high mass of AuNPs and the coupling between the LSPR of AuNPs and the SPR of gold thin film [252]. Qian et al. developed a fiber-optic SPR sensing system for the detection of miRNAs with PBA-AuNPs for signal output and amplification [253]. As presented in Figure 24, after the hybridization of capture DNA and target miRNA, the boronic acid groups on PBA-AuNPs specifically reacted with *cis*-diol groups in the ribose backbones of miRNAs, producing an amplified SPR signal.

## 7. Conclusions

Boronate-affinity materials have attracted substantial attention in recent years. A broad variety of boronic acid-based molecules and nanomaterials have been successfully prepared and used to develop various detection platforms, including electrochemistry, fluorescence, SERS, colorimetry, and others. The dissociation constant of the complex between a single boronic acid molecule and a *cis*-diol group changes from 10^−1^ to 10^−4^ M, which is lower than the immunoaffinity interaction (*K*_d_ < 10^−7^ M). The relatively low affinity may limit the applications of boronic acid molecules in real-sample assays. However, when boronic acid derivatives were loaded on nanomaterials, their binding strength toward *cis*-diol-containing species was significantly enhanced (*K*_d_ = 10^−5^–10^−10^ M) [254]. In addition, thanks to the reversible and selective sugar-responsive property of boronic acid, boronate-affinity materials have been used for in vivo labeling of *cis*-diol-containing species with physiological and pathological functions.

Despite the successful applications of boronate-affinity materials so far, there are still several challenges to be addressed. First, the influence of the geometric morphology and functional groups of nanomaterials on the pKa, affinity, and selectivity of boronic acid-based biosensors should be systematically investigated for theirpractical applications in real-time targeting glycoproteins and glycans on cancer cells or receptors. Although boronate-affinity MIPs have been successfully employed as receptors, the combination of other porous materials (MOFs and COFs) with boronic acid molecules may further enhance the properties of boronate-affinity materials and improve the detection performance of biosensors. Second, the synthesis of some boronate-affinity materials is always complex and time-consuming, which may limit their applications in the determination of *cis*-diol-containing biomolecules. It would be more attractive to synthesize such materials using a “one-step” method. For example, boronic acid derivatives could be used as precursors to synthesize fluorescent QDs by self-assembly or as ligands to prepare MOFs. Third, the integration of boronic acid-based biosensors with wearable and non-invasive devices may show promising applications in continuous metabolite monitoring in biofluids such as tears, saliva, breath, and sweat. The translation of such wearable and intelligent devices into industry and medicine may meet the need to significantly improve the quality of life of patients. Fourth, although well-developed biosensors with boronate-affinity materials have been widely used to detect *cis*-diol-containing species in laboratories, none of them can be translated to single-used bioreactors for in-line, at-line, or off-line bioprocess monitoring because of their low selectivity and stability. Therefore, more devices or technologies should be combined with boronic acid-based biosensors, especially special sterile adaptor technologies and appropriate sampling devices. We believe that this review will inspire others to design more practical biosensors based on boronate-affinity interactions.

## Data Availability

Not applicable.
